# Association Between the Body Mass Index and Prostate Cancer at Biopsy is Modified by Genetic Risk: A Cross-Sectional Analysis in China

**DOI:** 10.1097/MD.0000000000001603

**Published:** 2015-10-23

**Authors:** Gui-Ming Zhang, Yao Zhu, Hai-Tao Chen, Cheng-Tao Han, Fang Liu, Jian-Feng Xu, Ding-Wei Ye

**Affiliations:** From the Department of Urology, Fudan University Shanghai Cancer Center, People's Republic of China (GMZ, YZ, CTH, DWY); Department of Oncology, Shanghai Medical Colleague, Fudan University, People's Republic of China (GMZ, YZ, CTH, DWY); Fudan Institute of Urology, Huashan Hospital, Fudan University, People's Republic of China (HTC, FL, JFX); State Key Laboratory of Genetic Engineering, School of Life Sciences, Fudan University, People's Republic of China (HTC, FL, JFX); Center for Genetic Epidemiology, School of Life Sciences, Fudan University, People's Republic of China (HTC, FL, JFX); and Center for Cancer Genomics, Wake Forest School of Medicine, Winston-Salem, NC (JFX).

## Abstract

Herein, we aimed to examine whether the association of body mass index (BMI) with prostate cancer (PCa) at biopsy differs according to genetic susceptibility.

In a multicenter prospective cohort including 1120 men undergoing diagnostic prostate biopsy in China, we evaluated the interaction between BMI and genetic risk score (GRS) comprising 24 PCa-associated single nucleotide polymorphisms (SNPs), as well as a GRS consisting of 7 SNPs derived from an East-Asian population. The genetic risk was defined as low, intermediate, or high when GRS fell in the first, second, and third tertiles, respectively.

We observed a significant interaction between BMI and PCa GRS (*P*_interaction_ = 0.047), suggesting that the predictive value of BMI on PCa was strongly modified by genetic susceptibility. In men with high genetic risk, BMI was an independent predictor of PCa (odds ratio [OR] = 1.167, *P* = 0.008) after adjusting for conventional risk factors. The relationship between BMI and PCa risk diminished (*P* = 0.990) in men with low genetic risk. The interaction was more pronounced with the East-Asian GRS (*P*_interaction_ = 0.032), suggesting that the overall GRS interaction most likely occurs through genetic susceptibility in the East-Asian population.

Our results suggest that the predictive effect of BMI on the PCa risk is strongly modified by individual genetic susceptibility. The association is more positive among men with high genetic risk for PCa.

## INTRODUCTION

Although much lower than that in Western countries, the incidence of prostate cancer (PCa) in the People's Republic of China has been rapidly increasing over the past few decades.^1^ According to the Chinese Cancer Registry Annual Report (2012), PCa ranks as the 6th most prevalent cancer and the 9th leading cause of cancer-related mortality in men, especially in urban area.^2^ Altered lifestyle, lengthened life expectancy, and increased prostate-specific antigen (PSA) screening likely contribute in part to the increase in PCa diagnosis. Although the full etiology of PCa is not well understood, growing evidence supports an association between obesity and PCa risk.^3^ Obesity has also been proved to be associated with increased overall PCa mortality.^4^ Epidemiological surveys have also suggested the concordant trends of increased obesity and PCa incidence in the People's Republic of China. Through dysregulation of the insulin-like growth factor signaling pathway, as well as abnormal adipokine levels and a pro-inflammatory condition, obesity may contribute to PCa carcinogenesis.^5,6^

PCa is also a disease characterized by a strong hereditary background. Twin studies suggest that approximately half of all PCas can possibly be explained by hereditary genetic alterations.^7^ This hypothesis has been further supported by recent genome-wide association studies (GWAS), which have discovered numerous disease-associated traits. Although single nucleotide polymorphisms (SNPs) impart only a moderate effect on PCa, they can synthesize an additive genetic risk score (GRS), which has been demonstrated to significantly discriminate an individual's PCa risk over PSA alone. Aly et al found that the addition of GRS to a nongenetic model can avoid 22.7% of biopsies.^8^ Another Chinese study reported that GRS was an independent predictor of biopsy outcomes and might be helpful to determine the need for biopsy in patients within a “gray zone” of PCa risk.^9^

Because GRS may quantify the hereditary background of individuals, and PCa is thought to develop from an interplay between genetic and environmental factors, we raise an interesting question as to whether conventional predictors of PCa might express different effects in the context of genetic backgrounds. This hypothesis was suggested by a recent report which indicated that obesity, as evaluated by the body mass index (BMI), was an independent predictor of PCa only in patients with positive family history.^10^ To our knowledge, no study to date has investigated whether the association between BMI and PCa risk differs according to different genetic susceptibilities. Therefore, in this study, we examined the interaction of GRS with obesity on PCa risk at biopsy, aiming to identify the population that would benefit most from GRS.

## PATIENTS AND METHODS

### Study Subjects

This prospective maintained study included consecutive participants undergoing prostate biopsy from 2 tertiary medical centers in Shanghai from April 2012 to August 2014. Those who received repeated biopsies were counted as one subject. Transrectal ultrasound-guided biopsy using an 18 G needle was performed with at least 10 cores. The biopsy criteria required PSA > 4.0 ng/mL, or the presence of prostate nodules detected by ultrasound or digital rectal examination (DRE). We excluded those diagnosed with malignancies other than prostate adenocarcinoma.

A questionnaire was used to gather clinical information before biopsy. Age, BMI, smoking and drinking status, history of hypertension and diabetes, family history of PCa, PSA value, prostate volume, and pathological results were retrieved from our database. Smoking status (yes/no) was defined as current smokers or those who had ever smoked at least 100 cigarettes per year. Drinking status (yes/no) meant those ingesting any alcoholic beverage in the past 30 days.

The protocol was approved by the institutional research review boards of Fudan University Shanghai Cancer Center and Huashan Hospital, Fudan University, and written informed consent was acquired from all subjects before participation.

### DNA Extraction and Genotyping

Genomic DNA was extracted from fasting blood samples using the Qiagen Blood DNA Mini Kit (Qiagen Inc, Valencia, CA) and SNP genotyping was carried out by MassARRAY Iplex (Sequenom, Inc, San Diego, CA). For quality control, duplicates from 2 subjects and 2 water samples (negative controls) were placed in each 96-well plate. Eventually, the call rate was >98% for each of SNPs and the average concordance rate between samples was 100% among the duplicated quality controls.

### Statistical Methods

As previously reported,^9^ GRS was calculated for each participants based on their genotype of 24 SNPs (supplemental Table 1, http://links.lww.com/MD/A433), which have been confirmed to be associated with PCa risk in Chinese men.^11^ In addition, 7 SNPs that are specific in East-Asian populations were chosen to construct an East-Asian-specific GRS.^12^ In brief, GRS was calculated as follows: (1) we obtained the allelic odds ratio (OR) for each SNP from an external study,^12^ (2) we estimated the genotypic OR of each SNP from the allelic OR assuming a multiplicative model, (3) next we assessed the risk relative to the average risk in the population for each genotype based on genotypic OR and frequency in the Chinese population,^12^ (4) finally, we obtained GRS by multiplying the risks relative to the population of all SNPs.

Nonparametric Kruskal tests were used to compare differences in continuous variables, which are reported as medians with interquartile range, and chi-squared tests for categorical variables. The association between categorical variables and continuous variables were evaluated by LOESS plots and boxplots. Unconditional multiple logistic regression was performed to generate ORs and 95% confidence intervals (CIs). The *P*-values were 2-sided and *P* < 0.05 was considered statistically significant. All analyses were performed using R 3.0.1.

## RESULTS

A total of 1120 patients were included in our analyses, of which 486 (43.39%) patients were diagnosed with PCa. The general characteristics of all subjects stratified by GRS are listed in Table 1. As expected, we observed a significant difference in PCa probability between low and high GRS (35.3% vs 52.0%, respectively). Patients with high GRS were also more likely to harbor high PSA levels, abnormal DRE findings, and high-grade disease in biopsy specimen. The distribution of BMI was comparable among 3 GRS subgroups with a median value around 24, which is a typical value found in Chinese men. There was no significant association between BMI and age, PSA levels (log transformed), prostate volume, DRE findings, or GRS (log transformed; Supplementary Figure 1, http://links.lww.com/MD/A433). In addition, compared with those with negative biopsy outcomes, PCa patients were older, and had higher PSA levels, smaller prostate volume, and a higher incidence of abnormal DRE findings. No significant differences in BMI, smoking and drinking status, history of hypertension or diabetes, and family history of PCa were observed between groups (Supplementary Table 2, http://links.lww.com/MD/A433).

**TABLE 1:**
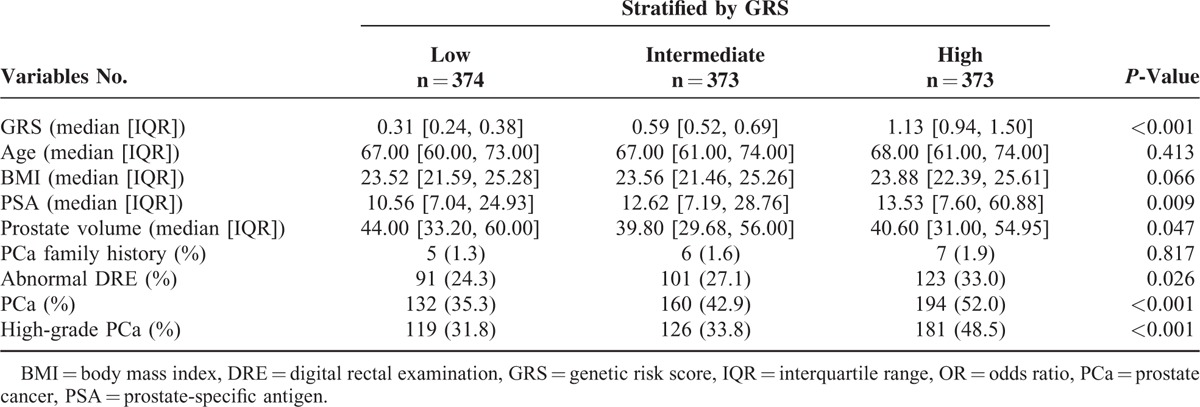
Baseline Characteristics of Subjects Stratified by Tertile of PCa GRS

**FIGURE 1:**
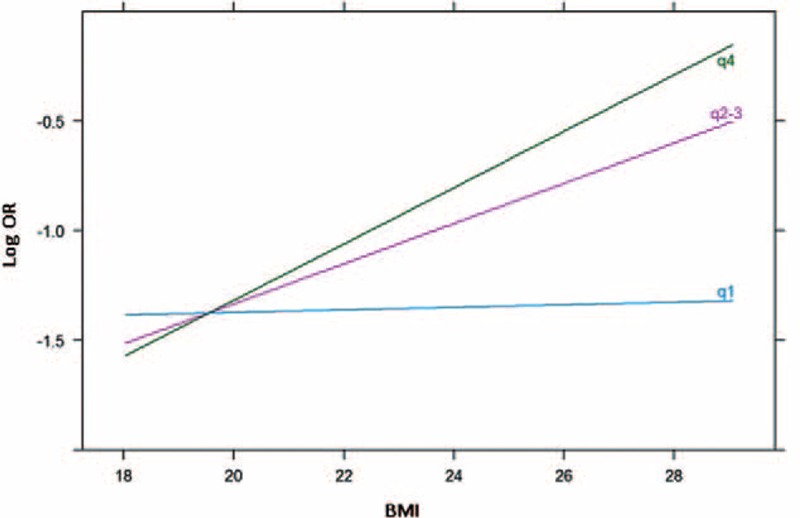
Association between BMI and OR (log transformed) stratified by total GRS. BMI = body mass index, GRS = genetic risk score, OR = odds ratio.

**FIGURE 2:**
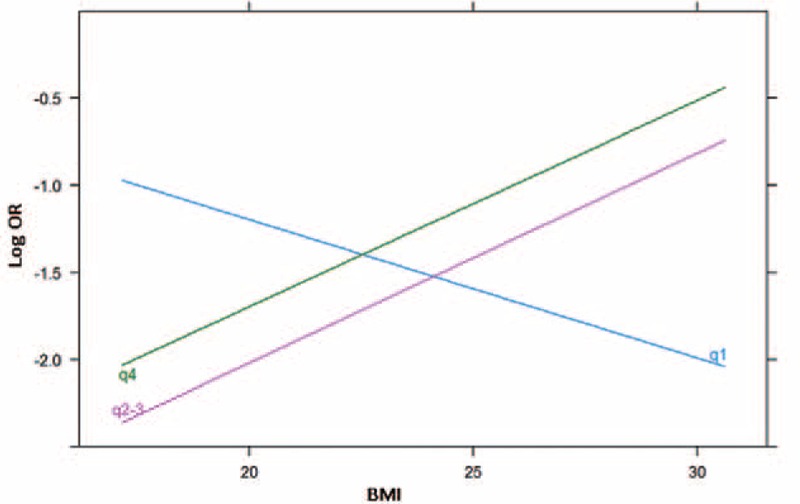
Association between BMI and OR (log transformed) stratified by East-Asian specific GRS. BMI = body mass index, GRS = genetic risk score, OR = odds ratio.

In the overall sample considering only main effects, we found that BMI was positively associated with PCa probability (OR_main_ = 1.076, *P* = 0.022; Table 2). The association between BMI and PCa risk was strongest in the high GRS group and weakest in the low GRS group. A significant interaction between PCa GRS with BMI on PCa risk was observed when the high GRS subgroup was compared with the low GRS subgroup (OR_interaction_ = 1.167, *P* = 0.047; Figure 1). The interaction was more pronounced when East-Asian-specific GRS was used to quantify genetic susceptibility. BMI was a strong predictor of PCa in patients with intermediate and high GRS. The interaction test yielded significant results when the intermediate or high GRS subgroup was compared with the low GRS subgroup (OR_interaction_ = 1.207, *P* = 0.015, and OR_interaction_ = 1.196, *P* = 0.021; Figure 2). Finally, we found a similar, albeit weaker, pattern of interaction of GRSs with BMI for high-grade disease risk (Supplementary Table 3, http://links.lww.com/MD/A433).

**TABLE 2:**
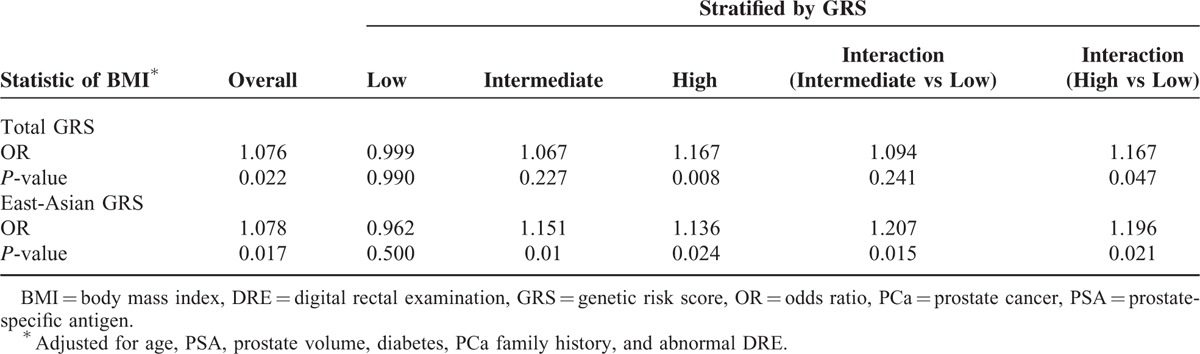
Adjusted OR and *P*-Value of BMI for PCa Probability According to GRS Strata and Test of Interaction

## DISCUSSION

In this multicenter cross-sectional prostate biopsy cohort study in Chinese men, we observed a significant interaction between BMI and PCa genetic risk on the probability of PCa at biopsy. Specifically, we found that the association between BMI and PCa risk was stronger in those with higher PCa genetic risk. In men with lower GRS, BMI failed to be an independent predictor of PCa. Furthermore, our results indicated that the interaction is likely driven through genetic risk loci specifically discovered in East-Asian populations. Taken together, our results suggest that the influence of BMI on PCa detection was, to a certain extent, modified by an individual's hereditary background.

Obesity has been demonstrated to be related to higher PCa risk and mortality.^3,13^ However, inconsistent conclusions among individual studies showed different effects of obesity on different cancer subtypes and tumors of different genetic origins.^10,14^ For example, lower levels of free testosterone were observed in obese men, which in turn was associated with decreased risk of localized/nonaggressive PCa and increased risk of advanced/aggressive PCa.^15–17^ Liang et al found that BMI was significantly associated with higher PCa risk only in those with a known family history.^10^ Another study reported a notably positive association between visceral obesity and aggressive PCa risk, mainly among Black men, but not non-Black men.^18^ Hence, the influence of obesity on PCa carcinogenesis may differ according to different genetic backgrounds. In our study, we found that BMI provided additional predictive value for PCa risk, and similarly, we also found a different influence of BMI on PCa prediction among men with different GRS. Although our results may not necessarily explain the causal role of obesity in PCa development, these findings suggest that BMI can be regarded as a predictor of positive biopsy outcomes, especially in men with higher GRS.

Previous studies have largely focused on the incremental value of GRS or environmental exposure for PCa prediction rather than on quantifying the interaction between genetic susceptibility and lifestyle factors. However, a better understanding of gene–environment interactions may provide us deeper insight into disease development, as well as more appropriate disease intervention. For example, Gu et al reported a different association of *ADIPOQ* genetic variants, which is an important molecular mechanism responsible for the link between obesity and PCa, and with PCa risks in the normal weight and overweight subgroups.^19^ Lifestyle intervention can reduce the risk of diabetes progression by ≥50% in high-risk individuals.^20^ In our results, the identified interaction between BMI and GRS suggests that environmental factors associated with obesity may modify inherited risk factors. Li et al observed that the effect of GRS on BMI could be remarkably attenuated by a physically active lifestyle.^21^ Although the exact mechanisms by which lifestyle and clinical factors influence genetic risk need further investigation, our findings suggest that obese men, especially those with high genetic risk, should pay more attention to PCa screening, and might benefit more from lifestyle alterations.

For the first time, we observed that the interaction between obesity and GRS was more evident when East-Asian GRS was used to quantify genetic susceptibility. Compared with patients in developed countries, PCa patients in the People's Republic of China are less likely to report a family history of PCa,^22^ as shown in our data. Furthermore, obesity is less prevalent in the People's Republic of China than in Western countries because of differences in lifestyle and caloric intake. All these genetic, environmental, and clinical factors might result in different modification effects of GRS on BMI in Chinese men. Obese men, compared with those of normal weight, are more likely to live an unhealthier lifestyle and may be more reluctant to screen for cancer. Furthermore, obesity has also been associated with lower circulating PSA levels and prostatic enlargement, making the detection of existing PCa more difficult.^23^ Thus, overweight and obese men with a higher GRS should be more actively screened, and hence PCa will be detected more frequently in this cohort.

Our findings may not only explain the heterogeneous results regarding BMI and PCa risk, but also generate the hypothesis that obesity–genetic interactions induce PCa carcinogenesis. Therefore, it is likely that men with strong hereditary backgrounds may be more susceptible to obesity-induced carcinogenesis. Using an East-Asian-specific GRS, we were able to create a genetic classification that shares a different hereditary background than Western patients. Interestingly, the East-Asian-specific GRS attenuated the modification effect of GRS on BMI. Therefore, East-Asian patients with ethnic-specific hereditary background were even more susceptible to obesity-related PCa. Furthermore, our findings highlight the importance of lifestyle intervention, especially weight control, in PCa prophylaxis.

There are several other strengths in our study. First, we implemented a multicenter prospective cohort study design, which may, to a large extent, reduce the potential reverse causation. Second, we used a PCa-related GRS defined from a relatively large panel of 24 SNPs that has been confirmed in Chinese men and validated its independent predictive value. Third, we performed a genetic dissection that may be implicated in this effect modification.

It is important to note the limitations of the present study. Although our research was conducted in 2 high-volume centers in eastern People's Republic of China, the sample size is still limited. In addition, Chinese patients were referred with specific symptoms, such as lower urinary tract symptoms or chronic prostatitis, indicating a high prevalence of confounding disease in this hospital-based cohort. Nevertheless, our study provided more implications toward clinical assessment of PCa risk and recommendations of prostate biopsies.

In summary, in this hospital-based prostate biopsy cohort, we found an interaction between genetic risk and obesity that affected the PCa risk, which appears to be driven nominally through East-Asian-specific genetic risk. BMI might provide independent predictive values for PCa detection on the basis of underlying genetic risk. Obese men, especially those with high GRS, should be educated on their risk of PCa to make better informed clinical decisions.
